# An Ensemble Successive Project Algorithm for Liquor Detection Using Near Infrared Sensor

**DOI:** 10.3390/s16010089

**Published:** 2016-01-11

**Authors:** Fangfang Qu, Dong Ren, Jihua Wang, Zhong Zhang, Na Lu, Lei Meng

**Affiliations:** 1 College of Computer and Information Technology, Three Gorges University, Yichang 443002, China; quff1128@163.com (F.Q.); wangjh@nercita.org.cn (J.W.); zhangzh_life@163.com (Z.Z.); luna199322@163.com (N.L.); 18230348796@163.com (L.M.); 2 Beijing Research Center for Agricultural Standards and Testing, Beijing 100097, China

**Keywords:** near infrared sensors, information processing, spectroscopy, variable selection, successive projections algorithm

## Abstract

Spectral analysis technique based on near infrared (NIR) sensor is a powerful tool for complex information processing and high precision recognition, and it has been widely applied to quality analysis and online inspection of agricultural products. This paper proposes a new method to address the instability of small sample sizes in the successive projections algorithm (SPA) as well as the lack of association between selected variables and the analyte. The proposed method is an evaluated bootstrap ensemble SPA method (EBSPA) based on a variable evaluation index (EI) for variable selection, and is applied to the quantitative prediction of alcohol concentrations in liquor using NIR sensor. In the experiment, the proposed EBSPA with three kinds of modeling methods are established to test their performance. In addition, the proposed EBSPA combined with partial least square is compared with other state-of-the-art variable selection methods. The results show that the proposed method can solve the defects of SPA and it has the best generalization performance and stability. Furthermore, the physical meaning of the selected variables from the near infrared sensor data is clear, which can effectively reduce the variables and improve their prediction accuracy.

## 1. Introduction

With the development of the agriculture and agricultural products processing industry (such as food, beverage, feed, tobacco, *etc.*), attention is not only on the product yield, but also the quality and safety of agricultural products. Wherein, liquor is a kind of agricultural product that is usually made from grain, wheat and sorghum by cooking, saccharification, fermentation, and distillation, and it has huge economic benefits. However, in commerce, the contents of alcohol in liquor is not standard, which seriously damages the interests of the consumers. A series of methods have been proposed to detect the contents of alcohol in liquor, such as using carbon nanotubes acoustic and optical sensors [1], co-immobilized peroxidase and alcohol oxidase in carbon paste [2], and rhythm and formant features [3]. However, most of these are classical chemical methods, the analysis process is complex, and the analysis period is long. Therefore, a fast and accurate method to detect the contents of alcohol in liquor, based on a near infrared (NIR) sensor [4,5], is urgently needed.

With the development of sensor technology, sensor information processing technology has received more and more attention [6,7]. The spectra retrieved from the near infrared (NIR) sensors have the potential to extract chemical information about the composition of a sample [8,9]. It has been widely used for the qualitative and quantitative analysis of complex products in agriculture. Alcohol concentration is one of the main quality and technical indicators in the production and sales of liquor, which can be detected quickly and accurately using an NIR sensor. However, NIR spectroscopy from NIR sensor mainly concerns the molecular absorption of multiplication and combination frequencies [10]. It not only reflects the chemical composition and content of the tested material, but also contains a spectrum response that is caused by many factors such as the temperature, surface texture, density, and uneven distribution of internal components of the measured object [11,12]. As a result, spectral information overlaps and has a high degree of collinearity. Accordingly, redundant information needs to be excluded from the complex spectral information before the useful information is extracted to improve prediction accuracy and efficiency while simplifying the model [13].

The successive projections algorithm (SPA) is a forward selection method that uses vector projection analysis in a vector space to minimize variable collinearity [14,15]. It can effectively eliminate the effects of redundant variables, singularity, and instability while reducing the number of variables and complexity of the model [16]. Hence, it can increase model speed and efficiency [17,18]. In addition, SPA selects effective wavelength, which has more physical meaning than the full spectrum of partial least squares (PLS) because it selects the variables with minimum collinearity directly from the original variables [19]. In contrast, PLS uses latent variables to extract useful information from the spectral data [20]. A latent variable is a linear combination of the original variables that can reflect the information better, but its physical meaning is not clear [21]. The advantages of SPA mean that it has been widely used in spectral variable selection. However, SPA has two disadvantages: (1) If the sample size of the calibration set is small, the samples for modeling are unrepresentative. Although the variable collinearity of the calibration set is minimized, on the validation set, an inappropriate selection of variables could mean that the prediction results are not satisfactory [22,23,24]. (2) SPA is an unsupervised variable selection method, therefore, the selected variables do not necessarily reflect the information of the measured component well [25,26].

To overcome these problems, an ensemble SPA variable selection method (EBSPA) based on a new variable evaluation index (EI) is proposed in this paper. First, using the bagging ensemble strategy, the bootstrap method is used to resample with replacement [27]. The union set of variables selected in parallel by SPA on different sample sets, bootstrap ensemble SPA (BSPA), is used for ensemble modeling to solve the problem of model instability caused by variable selection on small sample sets. Second, a new EI that is associated with the analyte is proposed to evaluate the importance of variables. The EI is used to sort the importance of the variables in the BSPA. Finally, the cross-validation PLS method is used to select the best subset of variables from the sorted variables, thus ensuring that the ultimately selected variables not only have low autocorrelation, but also have a certain crosscorrelation with the analyte. In this paper, we use three methods to establish the models of variables that are selected by EBSPA: the EBSPA multiple linear regression model (EBSPA-MLR), EBSPA PLS regression model (EBSPA-PLS), and EBSPA least-squares support vector machine model (EBSPA-LS-SVM). An experimental comparison of the proposed methods with traditional SPA and five other state-of-the-art methods, the Forward interval PLS method (FiPLS) [28], Backward interval PLS method (BiPLS) [28], elimination of uninformative variables method (UVE) [29,30], Monte-Carlo UVE (MC-UVE) [31,32], and competitive adaptive reweighted sampling (CARS) [33,34] is presented. The results show that, combined with MLR, PLS, and LS-SVM, the proposed EBSPA method can effectively reduce the number of variables while increasing model accuracy.

## 2. Materials and Data

### 2.1. Sample Preparation

Liquor and deionized pure water were used to exactly formulate 162 samples of 2 mL each. The concentrations varied from 4.5% to 85.0% in intervals of 0.5%. The 162 samples were divided into two groups using the sample set partitioning based on joint x-y distances (SPXY) method [35] with a ratio of 2:1. Thus, there were 108 and 54 samples in the calibration and validation sets, respectively. The calibration set was used for training the samples, and the validation set was used for testing the samples. Table 1 shows the statistical results of the alcohol content in the samples. Note that the concentration range of the validation set was included in the concentration range of the calibration set. Thus, it is compliant with modeling standards.

**Table 1:** Descriptive statistics for sample measurements.

Dataset	Number of Samples	Concentration Range (%)	Mean Value (%)	Standard Deviation
Calibration	108	0.045–0.850	0.419	0.2425
Validation	54	0.075–0.835	0.468	0.2266

### 2.2. Spectral Acquisition from NIR Sensor

An infrared spectrometer produced by PerkinElmer, Inc. (Waltham, MA, USA) was used for the experiments, which installs multiple sensors (e.g., DTGS and MCT) and supports fast and reliable sensor switch. The wavenumber ranged from 12,000 to 4000 cm^−1^. A total of 32 scans with a resolution of 4 cm^−1^ and interval of 2 cm^−1^ were performed. Thus, each spectrum had 4001 variables. The experimental instruments also included a PC and a manual pipette (Eppendorf, Germany). The spectrometer software used to collect the spectral data was Spectrum Version 10.4.1. The indoor temperature was kept at about 25 °C, and the humidity remained basically unchanged (less than 60%). Each sample was collected three times in parallel, and the final spectrum of the sample is the average of these three samples. To ensure the consistency of the measurement environment and manual operations, the background was scanned every 10 samples to eliminate drift.

### 2.3. Spectral Preprocessing

Different spectral processing methods have a different impact on model performance. The following methods were considered to determine which was best for all 162 samples: raw spectra without processing (RAW), multiplicative scatter correction (MSC), standard normal variable transformation (SNV), SNV plus the trend method (SNV+DT), Savitzky-Golay smoothing convolution (SG), sliding window smoothing (SW), first-order derivative (1-Der), and second-order derivative (2-Der) spectra methods. Table 2 presents the results calculated by the PLS model. As can be seen, SNV produced the best performance, achieving an R value of 0.9521 and RMSECV of 0.0715. 

**Table 2:** Modeling results of different processing methods.

Method	RAW	MSC	SNV	SNV + DT	SG	SW	1-Der	2-Der
R	0.8994	0.9325	0.9521	0.9444	0.8993	0.8991	0.9507	0.9512
RMSECV	0.1020	0.0845	0.0715	0.0769	0.1020	0.1020	0.0753	0.0771

**Figure 1:**
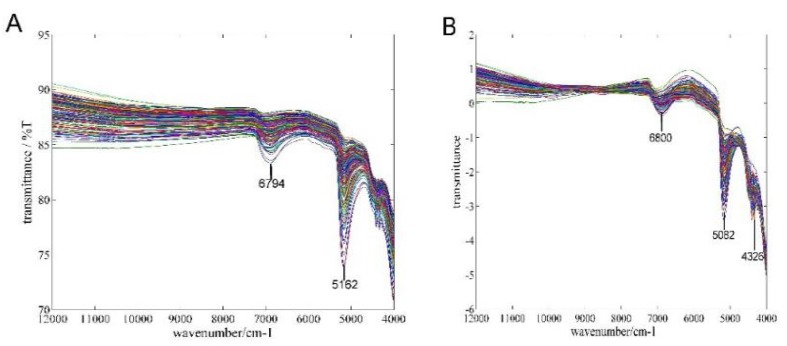
Spectra of samples: (**A**) RAW spectra and (**B**) SNV spectra.

Figure 1A depicts the NIR spectra of different concentrations of liquor. It shows the maximum absorption peaks are at 5162 cm^−1^, which mainly reflects the O–H stretching vibration, bending vibration, and a combination of C–H bending vibration of the absorption band [36]. These characteristic peaks have been widely used for the quantitative analysis of the alcohol content in liquor [37]. Figure 1B shows the spectra from NIR sensor that have been processed by SNV. The spectral absorption peaks have increased and are more obvious, making them more conducive to spectral analysis. Therefore, SNV was selected as the final processing method for the comparative experiments in this study. 

## 3. The Proposed Method

### 3.1. SPA

For spectral matrix $X_{N\times M}$ of the calibration set, where N is the number of samples, M is the number of variables, and H is the maximum number of the selected variables, the SPA algorithm is as follows:

1. Step 1: In the initial iteration t=1, a column vector $x_{j}$ is arbitrarily selected and denoted as $x_{k(0)}$, where k(0) is the starting position of the first selected variable. The location of the remaining columns are defined as s, where s={j,  1≤j≤M,  j∉{k(0),⋯,k(H−1)}}.
2. Step 2: Calculate the projection of the remaining column vectors $x_{j}$(j∈s) with respect to the orthogonal vector space that consists of the selected vectors $x_{k(t-1)}$:
  (1) $$\left\{ \begin{array}{l} P=I-\frac{x_{k(t-1)}{\left(x_{k(t-1)}\right)}^{T}}{{\left(x_{k(t-1)}\right)}^{T}x_{k(t-1)}}\\ x_{j}=Px_{j} \end{array}\right.$$
  where I is the identity matrix and P is the projection operator.
3. Step 3: Extract the variable that has the maximum projection value $\arg[\max(\left\|Px_{j}\right\|)]$, (j∈s), and add it to the set of selected variables.
4. Step 4: Let t=t+1. If t<H, return to Step 2 until t=H.

A crucial aspect of SPA is the selection of k(0) and H. Because there is collinearity between variables, the value of H generally cannot be too large. Otherwise, all of the projection values of the spectra will become zero [38]. For each selection of k(0), a method such as MLR or PLS is used to conduct the cross-validation analysis. To obtain the minimum value of the standard error of cross validation (RMSECV), the corresponding k(0) and the actual number of selected variables h(h≤H) are the final optimal choice.

### 3.2. EI

To ensure that the selected variables have both lower autocorrelation and some cross-correlation with the analyte, a new EI $w_{i}$ is introduced in this paper to select the best subset of variables, and is defined as

(2) $$w_{i}=\alpha_{i}\cdot p_{i}\cdot b_{i}$$

where $\alpha_{i}$ is the weight coefficient of the i-th variable. The order of the selected variables represents variable importance, which is sorted in descending order. The ordinal number of the variable corresponds to its weight, thus the more important variables have a larger weight. In the variable set that is obtained by multiple resampling, the weights of the same variable are summed when forming the union set. This further reflects the importance of recurring variables.

In addition, $p_{i}$ is the spectral purity value. It expresses the contribution of the i-th variable to the full spectrum. Larger values indicate greater contribution. Here, $p_{i}$ is defined as $p_{i}=\sigma_{i}/\mu_{i}$, where $\sigma_{i}$ is the standard deviation of the i-th variable and $\mu_{i}$ is its mean value.

Further, $b_{i}$ is the absolute value of the regression coefficient of the i-th variable. For the multivariate calibration model y=Xb+e, where y is the measured property vector, X is the spectral matrix, b is the regression coefficient vector, and e is the residual vector. The regression coefficient reflects the change of the spectral signal that is caused by the change of the unit concentration of the analyte. If $b_{i}$ is large, it indicates there is a good linear relationship between y and X.

Finally, $w_{i}$ combines the properties of $\alpha_{i}$, $p_{i}$, and $b_{i}$ in Equation (2). This equation is a more comprehensive evaluation of the variables. Therefore, selecting the variables that have large $w_{i}$ will help improve the prediction accuracy of the model [39].

### 3.3. EBSPA

In the EBSPA method that is proposed in this paper, a bootstrap method is used to obtain T sample sets from the original training set. SPA is then used to select variables from these sample sets. The invalid variables are removed from each sample set to obtain T sets of the selected variables. The union of these T sets without duplicated variables is then obtained. A new EI is used to evaluate the variables of the union set, and these variables are sorted in order of their importance. Finally, the PLS cross-validation method is used to select the final variables for modeling. The details of EBSPA are as follows:

1. Step 1: Set the number of iterations to T. Use the bootstrap method to randomly select samples with replacement from the calibration set. In each iteration, the number of picked samples is the same as the size of the calibration set. The set of selected sample is $S_{i},(i=1,\cdots ,T)$.
2. Step 2: Use SPA to select the variable subset $F_{i}$ from $S_{i}$.
3. Step 3: Let i=i+1. If i<T, then return to Step 1 to continue until the end of the iterations.
4. Step 4: Take union set $F_{i}(i=1,\cdots T)$ of the T sets of the selected variables, remove the repeated variables, and obtain the ensemble set of variables $F_{B}$.
5. Step 5: Calculate the importance of each variable in $F_{B}$ according to Equation (2), and arrange the EI values w in descending order.
6. Step 6: Use the PLS cross-validation method to successively accumulate the sorted variables starting with maximum w. When the minimum value of RMSECV is acquired, use the accumulated variables as the final selected set $F_{EB}$ for EBSPA modeling.

**Figure 2:**
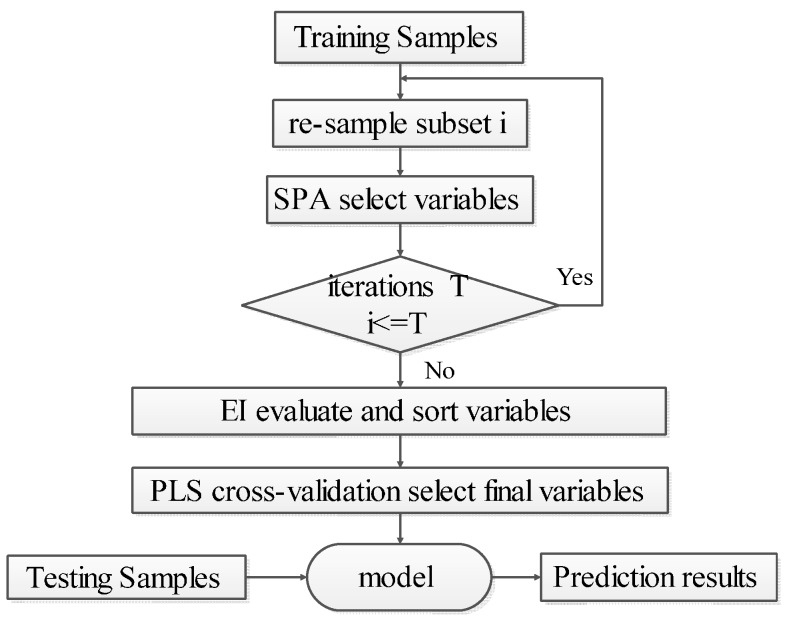
Flowchart of the proposed method.

Figure 2 outlines the framework of EBSPA. In this paper, new sample sets are obtained by multiple resampling. The characteristics of the bootstrap approach show that, in the original calibration set, some of the samples may be repeated several times, while others may never be selected at all. The ensemble method can increase the difference of the models by bootstrap resampling [40]. This ensemble strategy can be used to enhance the accuracy of small sample sizes when coupled with the calculation power of modern computing hardware. Furthermore, for the ensemble set of the variables, a new EI is proposed in this paper. The final valid variables associated with the measured substance are selected according to this index.

## 4. Experiments and Discussion

### 4.1. Parameter Selection

#### 4.1.1. Maximum Number of Selected Variables

The main parameter of both SPA and EBSPA is the maximum number of the selected variables H. When H is large, the projected effect changes and the amount of calculation is increased. When H is too small, the information of the selected variables is insufficient and the model will have poor accuracy. With the iterations of EBSPA set to 10, the prediction performances of SPA and EBSPA for H values of 10, 15, 20, 25, and 30 are listed in Table 3. 

Table 3 shows the prediction performance of EBSPA-MLR, SPA-MLR, EBSPA-PLS, and SPA-PLS. The results of EBSPA-MLR and EBSPA-PLS are better than those of SPA-MLR and SPA-PLS, which indicates that the proposed EBSPA method can effectively improve the prediction accuracy of the model. When H is 10, the results of all four methods are relatively poor. This may be caused by a lack of information of the selected variables. As a result, the models cannot achieve optimum results. When H is over 15, the results of SPA-PLS remain unchanged, and the final actual number of the selected variables h is 13. When H is over 20, the results of SPA-MLR are stable, and the final actual h is 17. Hence, the prediction performance of SPA does not continue to improve when H is greater than 20. On the contrary, it increases the computational cost of the model. 

Additionally, the experiments also compared EBSPA-LS-SVM and SPA-LS-SVM for the five values of H. A radial basis function was selected as the kernel function of LS-SVM. A grid search combined with leave-one-out cross-validation was used to determine the regularization parameter γ and kernel parameter $\sigma^{2}$ [41]. With a training set of 108 samples for modeling, the optimal SPA-LS-SVM parameters $\left(\gamma ,\sigma^{2}\right)$ were determined to be (5.278, 0.0039). Different values of H have no impact on EBSPA-LS-SVM and SPA-LS-SVM. The h, R, and standard error of prediction (RMSEP) of EBSPA-LS-SVM are 10, 0.9024, and 0.0882, respectively, regardless of the value of H. In addition, the h, R, and RMSEP of SPA-LS-SVM are 2, 0.8373, and 0.1123, respectively, for all values of H. Therefore, given the results of this comprehensive analysis, the parameter H was set to 20 in this study.

**Table 3:** Prediction performance for various H.

H	EBSPA-MLR		SPA-MLR		EBSPA-PLS		SPA-PLS	
	R2	RMSEP	R2	RMSEP	R2	RMSEP	R2	RMSEP
10	0.9587	0.0582	0.9183	0.0818	0.9548	0.0608	0.9154	0.0824
15	0.9599	0.0573	0.9129	0.0871	0.9611	0.0565	0.9542	0.0612
20	0.9614	0.0563	0.9269	0.0788	0.9654	0.0534	0.9542	0.0612
25	0.9625	0.0555	0.9269	0.0788	0.9671	0.0521	0.9542	0.0612
30	0.9445	0.0672	0.9269	0.0788	0.9677	0.0516	0.9542	0.0612

The maximum number of the selected variables is H, and R2 and RMSEP are the correlation coefficient and standard deviation of the validation set, respectively.

#### 4.1.2. Number of Iterations

**Figure 3:**
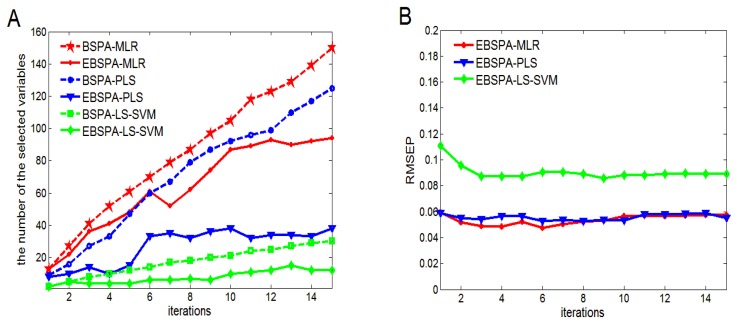
Model results for different values of T: (**A**) selected numbers of variables; and (**B**) RMSEP values.

For BSPA and EBSPA, there is another parameter, the number of iterations T. If T is too large, it will increase redundant information and the calculation of the models. If T is too small, it can have an effect on the ensemble strategy. Figure 3 depicts the results obtained for an initial number of iterations from 1 to 15, where the maximum number of selected variables is 20.

As can be seen in Figure 3A, as the number of iterations increases, the selected variables of BSPA increase. EBSPA uses EI to select variables once again that are based on BSPA. These variables increase then gradually stabilize. Figure 3B shows that the RMSEP of EBSPA decreases and then becomes stable. When T is small, the ensemble effect is not obvious. The poor results are caused by a lack of sufficient information for the EBSPA modeling. When T reaches a certain value, the variable information of EBSPA reaches saturation. The selected variables and prediction performance then remain stable. A comprehensive analysis of Figure 3, shows that the performance of EBSPA-MLR, EBSPA-PLS, and EBSPA-LS-SVM begin to stay stable as T reaches 9, 6, and 10, respectively. On the one hand, these results reflect the importance of EI. On the other hand, they show the stability of the proposed EBSPA method. In this paper, T is set to 10 in the following experiments, as it is a value that makes the model stable without too much additional computation.

### 4.2. Performance Analysis of Small Sample Sizes

To verify the effect of the ensemble strategy on the performance of SPA for small sample sizes, the SPXY method was used to select (27, 14), (54, 27), and (81, 41) samples from the original training and testing sets (108, 54) to form new sample sets. These three small sample sets are in line with modeling standards. Setting T=10 and H=20 with the ensemble method, the union set of the variables that were selected by SPA were used for BSPA modeling. The prediction performances of SPA and BSPA were compared for each of these four sample sizes.

**Figure 4:**
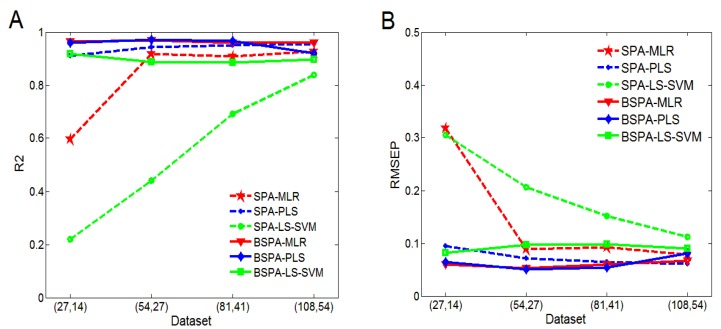
Experimental comparison of small sample sizes: (**A**) correlation coefficient and (**B**) RMSEP.

Figure 4 shows the results of the model predictions for the four groups of samples. It can be seen that the model performance of SPA is not stable at small sample sizes. Among the methods, the SPA-LS-SVM method is the most sensitive to the number of samples. The small number of selected variables leads to a lack of useful information for modeling. Hence, when the sample size is small, the prediction accuracy is poor. However, as the number of samples increases, the accuracy significantly improves. The prediction performance of SPA-MLR is poor for the sample set (27, 14), but its accuracy is quite good for the other three sample sets. The performance of SPA-PLS is good and stable at all four different sample sizes, the model is more accurate than that of SPA-MLR and SPA-LS-SVM, especially at sample set (27, 14), which implies insufficient samples.

As can be seen in Figure 4, the accuracy of BSPA combined with PLS is lower than that of SPA-PLS for the sample set (108, 54). Because the number of variables selected by SPA-PLS is large, redundant information may exist after variable integration. In the rest of the cases, the accuracy of BSPA is higher than that of the corresponding SPA. BSPA-MLR is more stable among these different sample sizes and has the best performance. Especially, the prediction performance of BSPA-LS-SVM is better for the sample set (27, 14) than the other three sample sets. In general, BSPA is not sensitive to the size of samples. Even for small sample sizes, BSPA can still achieve higher prediction accuracy. Therefore, these results show that the method based on BSPA can increase the specificity of samples to a certain extent, which can help solve the problem of small sample sizes and improve the accuracy of the model.

### 4.3. Performance Analysis of EI

EBSPA uses EI to evaluate the importance of the BSPA variables. The variables are sorted according to importance in descending order, and PLS cross validation is used to investigate the changes of RMSEP value with respect to the number of reserved variables. Finally, the minimum RMSEP is used to select the final variables of EBSPA. Table 4 lists the secondary selection results of EBSPA based on BSPA.

As Table 4 shows, for the four sample sets and three modeling methods, EBSPA can significantly reduce the number of variables in the final model by using the EI for the quadratic selection. On the one hand, it can effectively avoid the redundant information in BSPA that is caused by an excessive number of iterations. On the other hand, it can further improve the prediction accuracy of the model. The results show the feasibility and effectiveness of the proposed EI and further demonstrate the effectiveness of the ensemble variable selection for resolving the problem of small sample sizes. The use of EI can select the optimized variables that are important and relevant to the analyte, and it can compensate for the deficiency of SPA that arises from unsupervised variable selection. 

**Table 4:** Comparison of EBSPA and BSPA.

		(27, 14)	(54, 27)	(81, 41)	(108, 54)
BSPA-MLR	R2	0.9645	0.9682	0.9591	0.9592
	RMSEP	0.0599	0.0530	0.0594	0.0671
	$F_{B}$	117	116	107	105
EBSPA-MLR	R2	0.9687	0.9767	0.9606	0.9614
	RMSEP	0.0563	0.0455	0.0583	0.0563
	$F_{EB}$	50	57	40	87
BSPA-PLS	R2	0.9588	0.9716	0.9518	0.9192
	RMSEP	0.0644	0.0501	0.0643	0.0806
	$F_{B}$	119	83	94	92
EBSPA-PLS	R2	0.9704	0.9754	0.9665	0.9654
	RMSEP	0.0547	0.0467	0.0538	0.0534
	$F_{EB}$	20	28	36	38
BSPA-LS-SVM	R2	0.9166	0.8883	0.8843	0.8972
	RMSEP	0.0818	0.0973	0.0979	0.0904
	$F_{B}$	15	17	22	21
EBSPA-LS-SVM	R2	0.9204	0.9510	0.8985	0.9024
	RMSEP	0.0800	0.0633	0.0920	0.0882
	$F_{EB}$	13	10	11	10

Metrics R2 and RMSEP are the correlation coefficient and standard deviation of the validation set, respectively; $F_{B}$ and $F_{EB}$ are the number of variables in BSPA and EBSPA, respectively; and (m, n) denotes the sample set, where m and n are the number of samples of the calibration and validation sets, respectively.

### 4.4. Comparison of Different Spectral Variable Selection Methods

**Figure 5:**
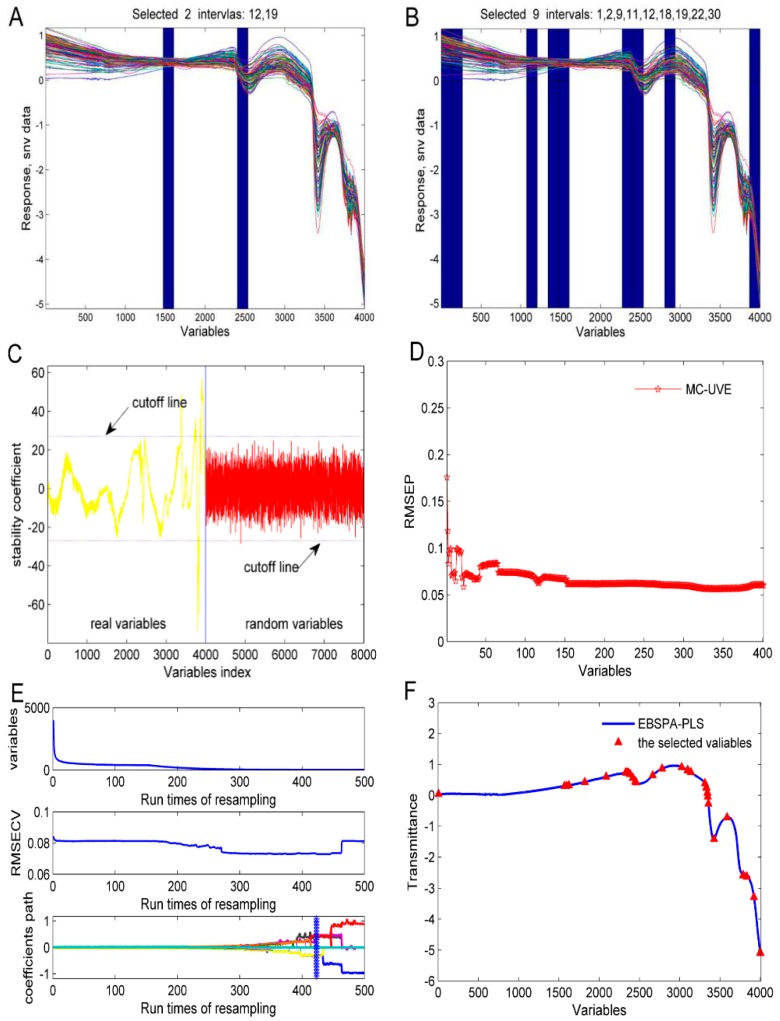
Variable selection: (**A**) FiPLS; (**B**) BiPLS; (**C**) UVE; (**D**) MC-UVE; (**E**) CARS; and (**F**) EBSPA-PLS.

To verify the validity of the EBSPA method proposed in this paper, we compared it with FiPLS, BiPLS, UVE, MC-UVE, and CARS, all efficient spectral variable selection methods. FiPLS and BiPLS are interval variable selection methods based on a spectral segmentation of PLS. UVE and MC-UVE are variable selection methods based on a leave-one-out cross validation and Monte Carlo sampling of the PLS regression coefficients, respectively. CARS imitates the “survival of the fittest” principle in Darwin’s evolution theory, and introduces an exponential decay function to control the variable retention rate for variable selection. These methods are all based on the PLS method, therefore, EBSPA-PLS is used for comparison. In this experiment, the spectra of FiPLS and BiPLS are divided into 30 intervals. The cutoff threshold of UVE was set to 0.9. The number of samples for CARS was 500. The number of iterations of EBSPA-PLS and MC-UVE were 10. These methods are modeled on the original calibration set (108, 54). The variable selection results are shown in Figure 5.

Figure 5A,B show the selected variable intervals for FiPLS and BiPLS, respectively. In these methods, the empirical values of spectral segmentation are usually 20–40 sections. When the full spectrum that has a total of 4001 variables is divided into 30 sections, for the first 19 intervals, each has 133 variables, and for the last 11 intervals, each has 134 variables. FiPLS selects two intervals of 266 variables and BiPLS selects nine intervals of 1199 variables, respectively. Figure 5C expresses the stability coefficient of the variable for each wavelength of UVE. The dotted lines are cutoff lines that are determined by the added random numbers. The variables between the two cutoff lines are considered to be uninformative variables that need to be eliminated, and, ultimately, 214 variables were reserved. Figure 5D presents the change of RMSEP with respect to the reserved variables. The RMSEP values are calculated at every 10 variables, from 1 to 4001. When 1571 variables are retained, the minimum value of RMSEP is 0.0616. Figure 5E describes the variable selection process of CARS. In the first 418 sampling models, RMSECV presents a decreasing trend, which indicates that the eliminated variables are useless. RMSECV then starts to increase, and it may eliminate useful variables. The minimum RMSECV is obtained at the 418th sampling, where there are 29 final variables selected. Figure 5F shows the 38 variables that EBSPA-PLS finally selected.

**Figure 6:**
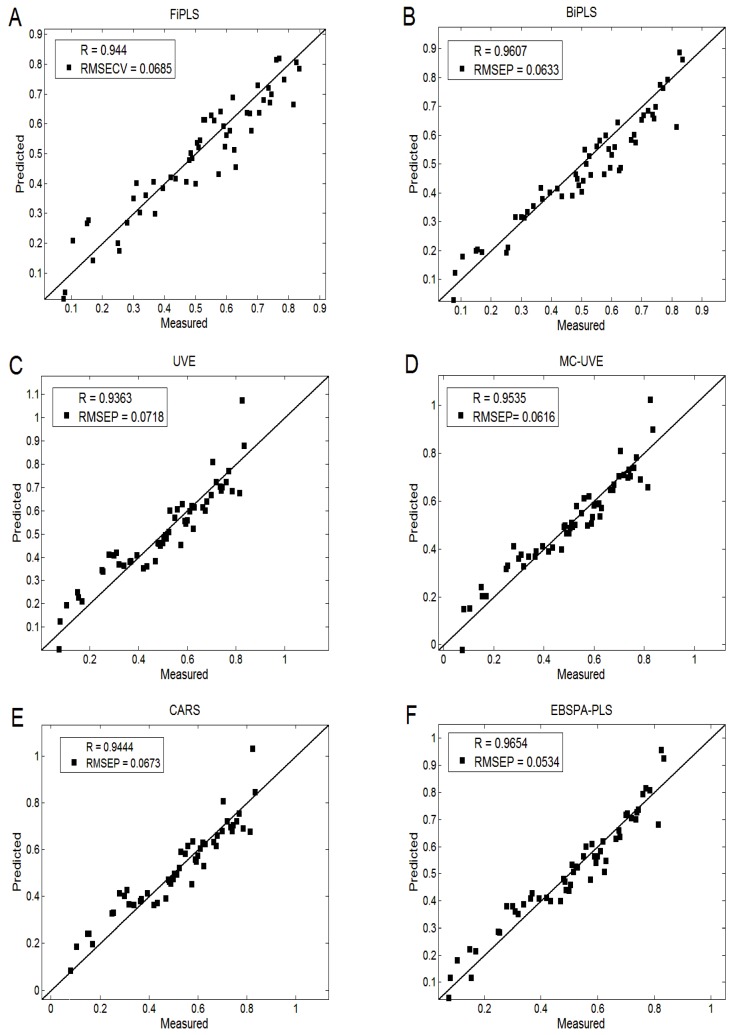
Comparison of regression rates: (**A**) FiPLS; (**B**) BiPLS; (**C**) UVE; (**D**) MC-UVE; (**E**) CARS; and (**F**) EBSPA-PLS.

Table 5 shows the performance of PLS without variable selection and the six variable selection models. It can be seen that the method proposed in this paper has the highest accuracy and its number of selected variables is small. This can effectively reduce the redundant information of variables, simplify the model, and improve its prediction accuracy. Figure 6 plots the corresponding regression rates. We can see that the sample points of EBSPA-PLS are more concentrated and closer to the regression line, which indicates that the prediction performance is better.

**Table 5:** Comparison of model performances.

Method	Calibration Set		Validation Set		Variable Numbers
	R1	RMSEC	R2	RMSEP	
PLS	0.9562	0.0707	0.9553	0.0605	4001
FiPLS	0.9696	0.0594	0.9440	0.0685	266
BiPLS	0.9711	0.0578	0.9607	0.0633	1199
UVE	0.9566	0.0704	0.9363	0.0718	214
MC-UVE	0.9536	0.0614	0.9535	0.0616	1571
CARS	0.9568	0.0702	0.9444	0.0673	29
EBSPA-PLS	0.9734	0.0523	0.9654	0.0534	38

Metrics R1 and RMSEC are the correlation coefficient and standard deviation of the calibration set, respectively; and R2 and RMSEP are the correlation coefficient and standard deviation of the validation set, respectively.

## 5. Conclusions

In this paper, the near infrared sensor has been implemented for obtaining the spectral data of liquor, and an ensemble successive project algorithm is proposed for variable selection and alcohol content detection in these liquor data. The proposed EBSPA can address two defects of SPA and improve the prediction accuracy of alcohol concentrations. The experimental results show that whether combined with linear MLR and PLS or nonlinear LS-SVM for modeling, the proposed EBSPA method can effectively address the disadvantages of SPA. It can simplify the model and improve prediction accuracy. In addition, when compared with FiPLS, BiPLS, UVE, MC-UVE, and CARS, the proposed EBSPA-PLS behaves better. Furthermore, it is shown that the use of NIR sensor and the proposed EBSPA can improve the performance of models with high prediction accuracy and stability, which can be applied for online and real-time detection of alcohol. It can also be effectively used to select variables and applied to NIR sensor data analysis in agriculture.
